# 

**Published:** 1978

**Authors:** 


### BRISTOL MEDICO-CHIRURGICAL JOURNAL

JULY/OCTOBER 1978
VOLUME 93 (iii/iv)
NUMBER 347/348

